# The investigation of construction and clinical application of image recognition technology assisted bronchoscopy diagnostic model of lung cancer

**DOI:** 10.3389/fonc.2022.1001840

**Published:** 2022-10-27

**Authors:** Yihong Deng, Yuan Chen, Lihua Xie, Liansheng Wang, Juan Zhan

**Affiliations:** ^1^ Department of Pulmonary and Critical Care Medicine, the Third Xiangya Hospital, Central South University, Changsha, Hunan, China; ^2^ Department of Computer Science, School of Informatics, Xiamen University, Xiamen, Fujian, China; ^3^ Department of Oncology, Zhongshan Hospital affiliated to Xiamen University, Xiamen, Fujian, China

**Keywords:** lung cancer, bronchoscopy, pathological type, image recognition technology, deep learning method

## Abstract

**Background:**

The incidence and mortality of lung cancer ranks first in China. Bronchoscopy is one of the most common diagnostic methods for lung cancer. In recent years, image recognition technology(IRT) has been more and more widely studied and applied in the medical field. We developed a diagnostic model of lung cancer under bronchoscopy based on deep learning method and tried to classify pathological types.

**Methods:**

A total of 2238 lesion images were collected retrospectively from 666 cases of lung cancer diagnosed by pathology in the bronchoscopy center of the Third Xiangya Hospital from Oct.01 2017 to Dec.31 2020 and 152 benign cases from Jun.01 2015 to Dec.31 2020. The benign and malignant images were divided into training, verification and test set according to 7:1:2 respectively. The model was trained and tested based on deep learning method. We also tried to classify different pathological types of lung cancer using the model. Furthermore, 9 clinicians with different experience were invited to diagnose the same test images and the results were compared with the model.

**Results:**

The diagnostic model took a total of 30s to diagnose 467 test images. The overall accuracy, sensitivity, specificity and area under curve (AUC) of the model to differentiate benign and malignant lesions were 0.951, 0.978, 0.833 and 0.940, which were equivalent to the judgment results of 2 doctors in the senior group and higher than those of other doctors. In the classification of squamous cell carcinoma (SCC) and adenocarcinoma (AC), the overall accuracy was 0.745, including 0.790 for SCC, 0.667 for AC and AUC was 0.728.

**Conclusion:**

The performance of our diagnostic model to distinguish benign and malignant lesions in bronchoscopy is roughly the same as that of experienced clinicians and the efficiency is much higher than manually. Our study verifies the possibility of applying IRT in diagnosis of lung cancer during white light bronchoscopy.

## 1 Introduction

Lung cancer is one of the malignant cancer types with second highest morbidity and top mortality around the world, which seriously threatens human’s health. According to GLOBOCAN statistics, there were about 2.2 million new lung cancer cases worldwide in 2020, and the number of deaths reached 1.8 million (1). The high mortality rate of lung cancer is mainly due to lack of specificity in its early clinical manifestations. Most lung cancer patients are in advanced stages when detected, which seriously affects the survival and prognosis (2). Therefore, improving the detective methods of lung cancers will help to increase the detection rate, improve the follow-up diagnosis and treatment, thus reduce mortality (3).

The diagnosis of lung cancer requires pathological evidence, and bronchoscopy is currently one of the most common method for obtaining lung tissue specimens (4, 5). It can not only provide objective basis for subsequent therapies through biopsy, brushing and lavage, but also directly observe the location and shape of lesion in bronchial lumens to better understand the degree of lesion. If necessary, local interventional therapy can be performed through bronchoscopy (6). It has been widely used by pulmonologists for the diagnoses and treatments of pulmonary diseases.

The appearances of lung cancer under the microscope are sometimes hard to differentiate with severe chronic inflammation, tuberculosis or other lesions. During inspection, physician makes a preliminary judgment on the lesion and conducts brush examination or biopsy on the suspicious parts for pathological examination. Inappropriate operations not only increase financial burden of patients, but also cause bleeding emergencies (7). Moreover, insufficient recognition of malignant lesions will delay the opportunities for early diagnoses and therapies. Respiratory endoscopists’ abilities to judge the nature of lesions are related to skills and experiences, which are somewhat subjective. The probability of accurate judgments made by beginners and junior endoscopists is far lower than experienced senior endoscopists. Additionally, long time and large-scale examinations lead to missed or false judgements due to fatigue.

Recently, with the development of artificial intelligence(AI), IRT has also been investigated and applied extensively in the medical field. Through the established deep learning model, it can extract and learn the deep features of target image automatically, thus complete tasks such as target lesions detection, segmentation, classification and prediction (8). IRT has achieved important results in assisting the diagnosis of breast cancer, skin cancer, diabetic retinopathy and other diseases (9–11). Its use in assisting medical endoscopy in recognition of lesions is currently mainly applied in digestive endoscopy, such as the identification of Barrett’s esophagus, early gastric cancer, differentiation of colon polyps and etc (12–14). It has been verified to have higher efficiency, sensitivity and specificity than ordinary endoscopists. In the diagnosis of lung cancer, IRT based on deep learning has been used in the differentiation of benign and malignant lung nodules on images, pathological classification of lung cancer tissue slices (15, 16). The technology applied in identification of lung cancer lesions in most current studies is under fluorescence bronchoscopy, which is rarely used in white light bronchoscopy (17). And there is a lack of comparison with clinicians.

Therefore, we developed a bronchoscopy-assisted diagnostic system based on deep learning methods to determine initially benign and malignant lesions. The system model was constructed by training bronchoscopy images of 666 lung cancer patients and 152 non-cancerous controls. In addition, we tried to classify the pathological types of lung cancer through this diagnostic model, aiming to explore the value and significance of AI IRT to assist in the diagnosis of malignancies during white light bronchoscopy.

## 2 Methods

### 2.1 Preparation of training and test images

#### 2.1.1 Image acquisition of malignant lesion

**Figure 1:**
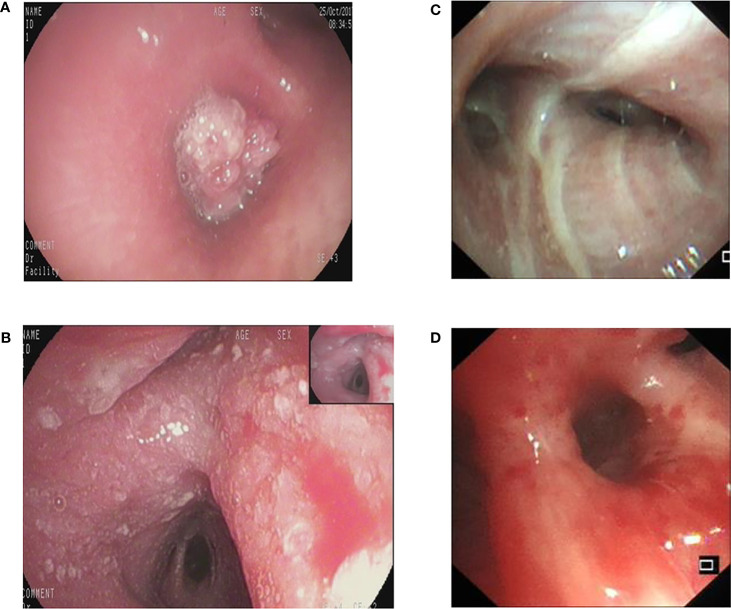
The different bronchoscopic manifestations of lung cancer. **(A)**: proliferative type; **(B)**: infiltrative type; **(C)**: stenotic type; **(D)**: inflammatory type.

Retrospectively obtained the bronchoscopic images of inpatients examined at the Bronchoscopy Center of the Third Xiangya Hospital, Central South University from Oct.01 2017 to Dec.31 2020. The inclusion criteria were the images showing lesions that were confirmed as lung cancers by pathological examinations through biopsies or brushing. The exclusion criteria were as follows:1). Images of poor quality caused by halo, defocus, mucus or bleeding. 2). Blurred images resulted from long distance. The bronchoscopic manifestations were divided into 4 types: proliferative, infiltrative, stenotic and inflammatory (**Figure 1**).

#### 2.1.2 Image acquisition of benign lesion

Retrospectively obtained the bronchoscopic images of inpatients examined at the Bronchoscopy Center of the Third Xiangya Hospital, Central South University from Jun.01 2015 to Dec.31 2020. The inclusion criteria were as follows: 1). The mucosal lesions were obvious. 2). There was no malignant lesion identified by pathological examination. 3). The clinical process of the lesion was clearly benign. The exclusion criteria were listed as: 1). There was only slight redness, edema and mild inflammation in mucosa. 2). Radiological evidence indicated malignant diseases in the lung or other organs.

### 2.2 Experimental methods and details

Based on the deep learning framework Pytorch of the FaceBook AI Research Institute, a series of preprocessing operations were performed on the data set, and then the processed training set data was input into the deep learning model ResNet101 to train the model (18, 19). During training, gave each picture its corresponding label, extracted the features through the neural network, and continuously optimized the model. After each epoch of training, the current model effect was evaluated through the validation set data. After all training is completed, the final effect of the model was evaluated through the test set data. Since multiple images were included in each case,when using the diagnostic model to predict the category of cases, images from different angles of the same lesion may be predicted as the opposite result. We predict each picture of the same case, and get the probability values belonging to a certain category,and add the prediction probability values corresponding to the same class. Finally, the category to which the maximum probability belongs was selected as the final case forecast result.

#### 2.2.1 Benign and malignant classification

Data preprocessing: The experimental data preprocessing can be roughly divided into two parts: 1) Divided the 2238 images of benign and malignant lesion data in bronchoscopy into the training, validation, and test set according to the ratio of 7:1:2. The number of cases and pictures is respectively 572 and 1552 in training set, 82 and 219 in verification set,164 and 467 in test set. 2) Changed the size of the data picture to 256 × 256 pixels, then cropped an image of 224 × 224 pixels in the center of the changed image, and finally normalized the data to map the pixel value of the picture between 0 and 1.

**Figure 2:**
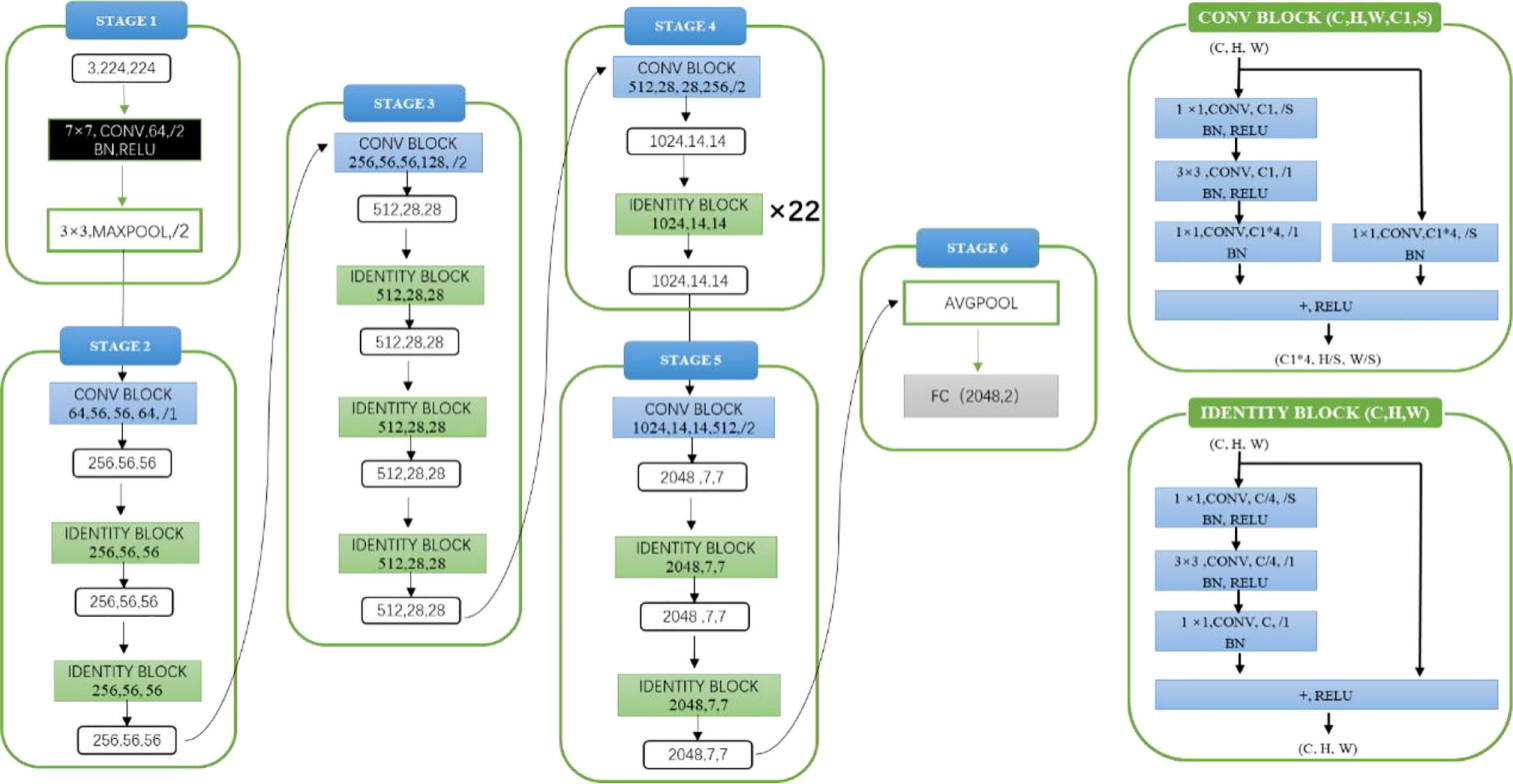
ResNet101 Network Structure.

Model architecture and experimental details: The experimental model architecture used ResNet101 (as shown in **Figure 2**). ResNet101 is one of the models of the ResNet series. ResNet is a classic network in the current image classification field and consists of a series of residual blocks. Its emergence resolves the problem of gradient disappearance and gradient explosion, as well as the network degradation caused by the number of network layers deepening to a certain extent. Before model training, the parameters of pre-training model were loaded as initialization parameters. The pre-training model was trained on the ImageNet data set. Loading the parameters of pre-training model was conducive to improving the generalization of the model and can improve the convergence rate of the model during gradient descent. In the process of model training, the loss function was used to judge the gap between the predicted value of the model and the label. The loss function used in this experiment was the cross-entropy loss function, the optimizer used Adam, the initial learning rate was 1×10^-4 and the batch size was 10.

#### 2.2.2 Pathological classification

Data preprocessing: The experimental data preprocessing can be roughly divided into two parts: 1) Divided the malignant lesion data (except the part whose pathological type could not be determined) into the training, validation and test set. The number of cases and pictures is respectively 433 and 1146 in training set, 62 and 151 in verification set, 124 and 291 in test set. 2) Changed the size of picture to 256 × 256, and cropped an image of 224 × 224 pixels in the center of changed image, and finally normalized the data to map the pixel value of the picture between 0 and 1. Additionally, during the preprocessing, the adenocarcinoma training data was horizontally flipped with a probability of 50% to enhance the data.

Model architecture and experimental details: The experimental model architecture used ResNet101 (as shown in **Figure 2**). Before model training, the parameters of the pre-training model (ImageNet) were also loaded as initialization parameters. The loss function used in the experiment is the cross-entropy loss function, the optimizer uses Adam, the initial learning rate was 1×10^-4, and the batch size was 10.

### 2.3 Outcome indicators

For benign and malignant classification models, calculate receiver operating characteristic (ROC), AUC, accuracy (ACC), specificity (Sp), sensitivity (Sn), positive predictive value (PPV) and negative predictive value (NPV) to evaluate the performance of the model. For pathological classification models, calculate the overall classification accuracy and recognition sensitivity of SCC, AC and small cell lung cancer (SCLC).

### 2.4 Comparison with clinicians

We invited 9 clinicians to diagnose the same test set images in diagnostic model. According to the experience of bronchoscope operation, clinicians were divided into senior, junior and novice group. The novice group included three master or doctoral students in respiratory medicine, who had little practical experience in bronchoscopy and was tested after simple training. The junior and senior group was respectively composed of three doctors with 1-3 years and over 10 years bronchoscope operation experience. The three groups independently diagnosed the same test set unaware of pathological results and clinical information about the cases.

### 2.5 Statistical analysis

The Mcnemar test was used to compare differences in the accuracy, sensitivity and specificity of the diagnostic model and clinicians. The Fleiss kappa coefficient was used to evaluate the variability between the clinicians and diagnostic model. All calculations were performed using SPSS 26.

## 3 Result

### 3.1 Clinical information

A total of 666 cases of malignant lesions and 152 cases of benign lesions were included in this study. In malignant group, there were 312 cases of lung SCC, 178 cases of lung AC, 129 cases of SCLC and 47 cases of others (41 cases of cancer with unknown pathological classification, 4 cases of adenosquamous cell carcinoma and 2 cases of neuroendocrine carcinoma) (**Table 3**). In benign group, there were 120 cases of infectious lesions (52 cases of pulmonary tuberculosis, 48 cases of community-acquired pneumonia, 9 cases of lung abscess, 8 cases of bronchiectasis, 3 cases of aspiration pneumonia), 8 cases of chronic bronchitis, 7 cases of obsolete tuberculosis, 4 cases of pulmonary contusion, 3 cases of interstitial pneumonia and 2 cases of tracheomalacia and 8 cases of others (hamartoma, pneumoconiosis, ossifying bronchitis, changes after pneumonectomy, pulmonary aspergillosis, tracheal polyp, tracheal diverticulum, granulation hyperplasia after tracheotomy) (**Table 1**). The characteristics of malignant and benign cases were shown in **Table 2**.

**Table 1:** The characteristics of malignant and benign group.

	malignant	benign
Number of people	666	152
Gender		
male	559 (83.9%)	75 (49.3%)
female	107 (16.1%)	77 (50.7%)
Age		
median	63 (30-84)	62 (16-83)
smoking history		
smoking	458 (68.8%)	40 (26.3%)
non-smoking	202 (30.3%)	111 (73.0%)
unknown	6 (0.9%)	1 (0.7%)

**Table 2:** Bronchoscopic manifestations of different pathological types of lung cancer (%).

	Proliferative	Infiltrative	Stenotic	Inflammatory	total
SCC	210(67.3)	70(22.4)	26(8.3)	6(1.9)	312
AC	48(27.0)	87(48.9)	37(20.8)	6(3.4)	178
SCLC	56(43.4)	53(41.1)	18(14.0)	2(1.6)	129
Other	22(46.8)	15(31.9)	8(17.0)	2(4.3)	47

### 3.2 Bronchoscopic manifestations of different pathological types of lung cancer

As shown in **Table 3**, proliferative lesion was the main type in malignant group, followed successively by infiltrative, stenotic and inflammatory lesion. Proliferative lesion was most common in patients with SCC, accounting for 67.3%. Invasive lesion was most common in patients with AC, accounting for 48.9%, followed by proliferative lesion, accounting for 27.0%. There was no significant difference in the proportion of proliferative and invasive lesions in SCLC group.

**Table 3:** Clinical distribution of benign lesions.

	Number of cases
Infectious lesions	120
Chronic inflammation of bronchus	8
Obsolete tuberculosis	7
Pulmonary contusion	4
Interstitial pneumonia	3
Tracheomalacia	2
Others	8
Total	152

### 3.3 The test result of diagnostic model

#### 3.3.1 Test results of differentiating benign and malignant lesions by diagnostic model

**Figure 3:**
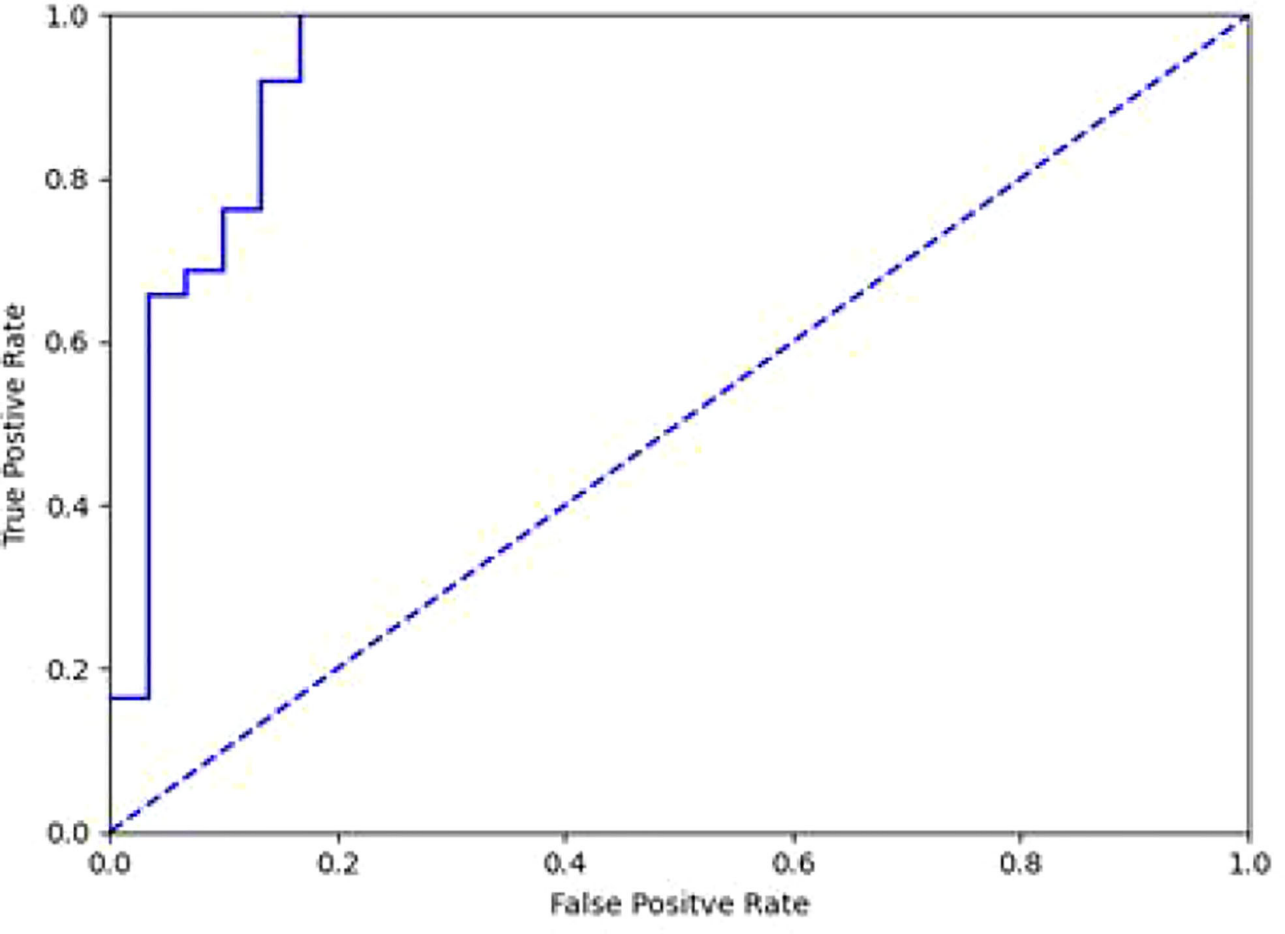
ROC curve of differentiation between benign and malignant lesions in diagnostic model(AUC=0.940).

It took 30 seconds to diagnose patients in the test set. **Table 4** shows the overall accuracy of model is 0.951. 131 of 134 lung cancer lesions were correctly identified, with a sensitivity of 0.978. 25 out of 30 benign lesions were correctly diagnosed with a specificity of 0.833, a positive predictive value of 0.963, a negative predictive value of 0.893, and an AUC value of 0.940(**Figure 3**). The details of 8 lesions with wrong classification by model were shown in **Table 5**, **Figure 5** and **Figure 6**.

**Table 4:** The benign and malignant differentiation test result of diagnostic model.

	No. of cases	ACC	Sn	Sp	PPV	NPV	AUC
malignant	134	0.951	0.978	0.833	0.963	0.893	0.940

**Table 5:** Cases of misclassification in diagnostic model.

Clinical diagnosis	Model Judgement	Lesion site	Form
Cancer (unknown type)	benign	Opening of upper lobe of left lung	Inflammatory
Adenocarcinoma	benign	Apical segment of upper lobe of left lung	Infiltrative
Adenocarcinoma	benign	Right middle bronchus	Proliferative
Changes after tracheotomy	malignant	Upper trachea	Proliferative
Obsolete tuberculosis	malignant	Dorsal segment of lower lobe of right lung	Stenotic
Pulmonary infection	malignant	lower lobe of right lung	Infiltrative
Pulmonary infection	malignant	lower lobe of right lung	Infiltrative
Pulmonary infection	malignant	Dorsal segment of lower lobe of left lung	Infiltrative

#### 3.3.2 Differentiation of lung cancer pathological types by diagnostic model

**Figure 4:**
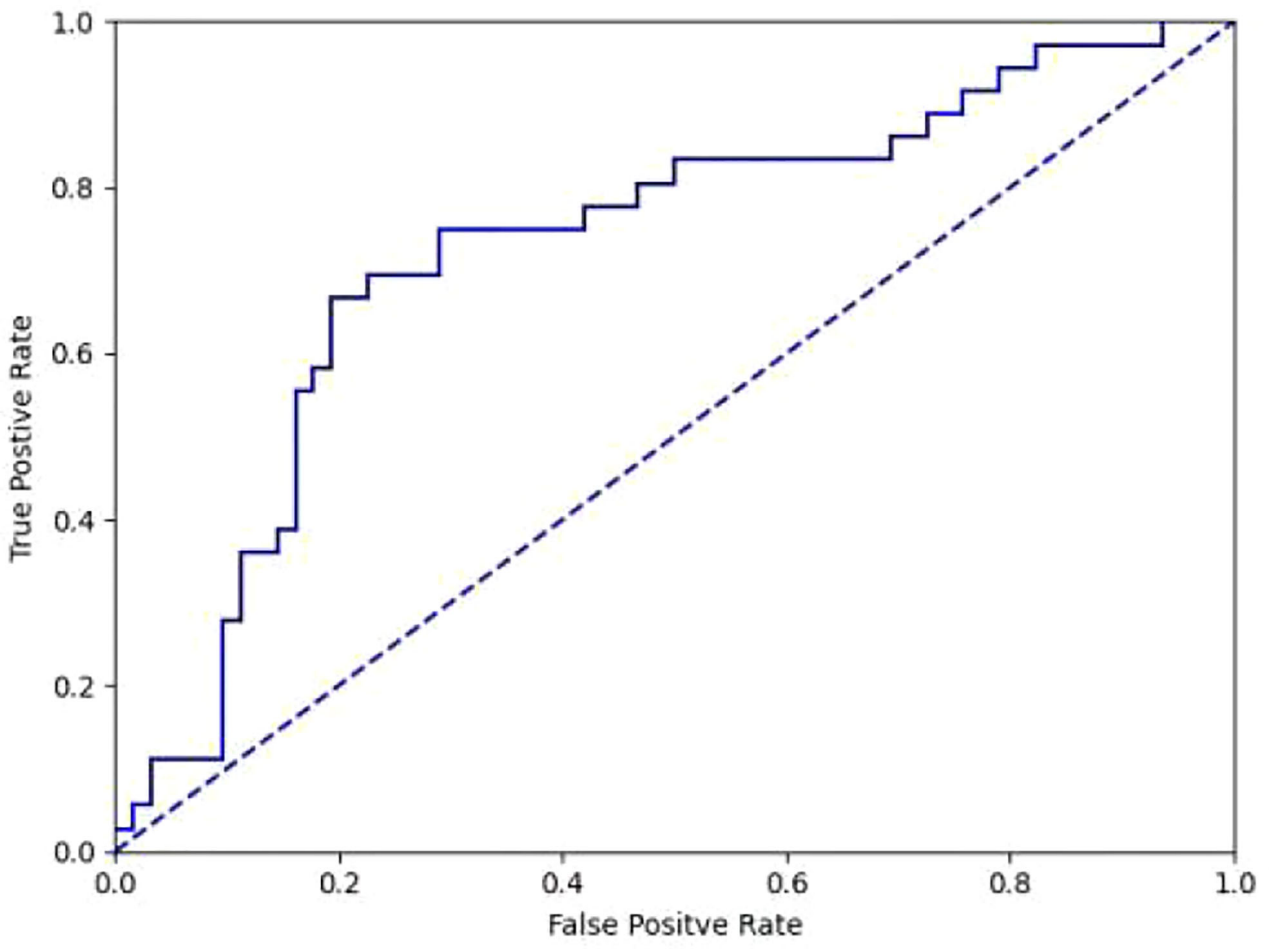
ROC curve of differentiation between SCC and AC in diagnostic model(AUC=0.728).

We attempted to classify 124 cases of SCC, AC, and SCLC in the test set, and obtained an overall accuracy of 0.605 (75/124), among which 0.710 (44/62) for SCC, 0.500 (18/36) for AC, and 0.500 (13/26) for SCLC. Considering that SCC is dominated by proliferative lesions while AC is most common as infiltrative lesions, we classified SCC and AC, which obtained the overall accuracy of 0.745, among which 0.790 for SCC and 0.667 for AC. The AUC value is 0.728 (**Figure 4**).

#### 3.3.3 Comparison of diagnosis results between clinicians and diagnostic model

As shown in **Table 6**, the diagnostic performance of the model was similar to that of 2 clinicians in senior group (*P >*0.05). Its accuracy and sensitivity were higher than those of novice and junior groups (*P <*0.05).

**Table 6:** Comparison between diagnostic model and clinicians in differentiating benign and malignant lesions.

	ACC	Sn	Sp	PPV	NPV
model	0.951	0.978	0.833	0.963	0.893
Senior 1	0.915	0.963	0.700	0.935	0.808
Senior 2	0.921	0.948	0.800	0.955	0.774
Senior 3	0.884^*^	0.903^*^	0.800	0.953	0.649
Junior 1	0.835^*^	0.851^*^	0.767	0.942	0.535
Junior 2	0.854^*^	0.896^*^	0.667	0.923	0.588
Junior 3	0.872^*^	0.888^*^	0.800	0.952	0.615
Novice 1	0.793^*^	0.881^*^	0.400^*^	0.868	0.429
Novice 2	0.774^*^	0.799^*^	0.667	0.915	0.426
Novice 3	0.720^*^	0.776^*^	0.467^*^	0.867	0.318

*means significant difference compared with diagnostic model (P<0.05).

The comparison of consistency between clinicians in each group and model is shown in **Table 7**. The consistency (kappa = 0.658) between Senior group and model was higher than that (kappa = 0.609) between junior group and model. There was a poor consistency between novice group and model(kappa=0.395).

**Table 7:** Consistency test between three groups with different experience and diagnostic model.

	senior and model	junior and model	novice and model
Fleiss kappa value	0.658	0.609	0.395
*P* value	0.000	0.000	0.000

## 4 Discussion

With the progress of precision medicine and medical imaging technology, the status of various medical images in disease diagnosis becomes more and more important. The demand for optimization and simplification of image diagnosis process is also increasing. The subjective process can be quantified and reproduced by IRT. The features are automatically learned from training images using deep learning. Even some image information that cannot be detected by humans can be identified, thereby helping doctors optimize clinical decision-making (20). In respiratory system, it has been more successfully used in identification of tuberculosis, pneumothorax, pneumonia, and pulmonary nodules on X-ray or CT images (21–23). In recent years, applying IRT to assist in lesion identification under endoscopy has become another hot topic, and there have been many studies in digestive endoscopy, laryngoscopy, and colposcopy (24–26). The technology is mainly used for identification of lung cancer in fluorescence bronchoscopy, but its application in white light bronchoscopy is scarce. Tan and others used sequential fine-tuning to optimize deep learning method, and then obtained an overall accuracy of 0.820 in making distinction of lung cancer, tuberculosis and normal tissues during white light bronchoscopy (27). In our study, we increased the types of benign lesions, used deep learning algorithms to distinguish lung cancer lesions under white light bronchoscopy from a variety of benign lesions. We also attempted to judge pathological types of lung cancer for the first time. In addition, most of previous studies under bronchoscopy lacked comparison with diagnostic performance of clinicians, and our investigation supplemented this shortage.

To discriminate benign and malignant lesions, we use ResNet101 residual network to train images. The residual block inside the network reduces information loss during process of feature extraction and alleviates the gradient disappearance problem caused by increase of deep learning layers. The final classification accuracy was as high as 0.951. Finkšt et al. used machine learning to classify benign and malignant tumors under fluorescence bronchoscopy, and finally obtained an accuracy of 0.958, which is comparable to our results (28). Compared to Finkšt’s test, the deep learning approach we employed avoids the complexities of manual feature extraction and classification framework design with traditional machine learning algorithms.

**Figure 5:**
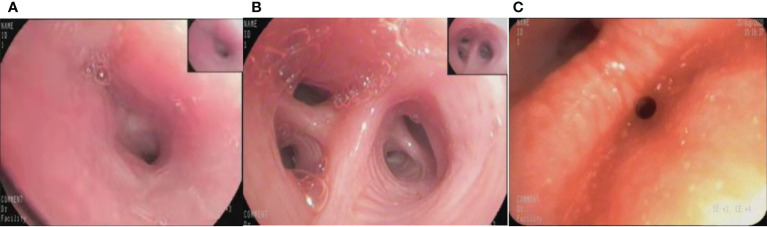
The misclassified malignant lesions. **(A)**: lung cancer with inflammatory manifestations **(B)**: lung adenocarcinoma with infiltrative manifestations **(C)**: lung adenocarcinoma with proliferative manifestations.

**Figure 6:**
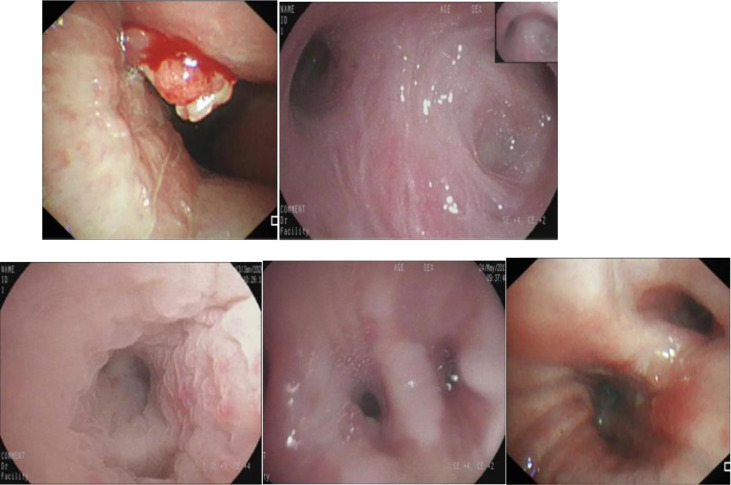
The misclassified benign lesions.

The diagnostic model successfully identified 131 of 134 lung cancer patients, with a sensitivity of 0.978.Among the 3 malignant lesions that were misclassified, one was a lung cancer lesion of unknown pathological classification with inflammatory manifestations (**Figure 5A**). In clinical practice, such inflammatory lesions of lung cancer under bronchoscope are difficult to identify even by experienced experts.The second case was an adenocarcinoma with infiltrative manifestations (**Figure 5B**), the lesion occupies a very small area of the whole picture (only about 16%),and because of factors such as reflection and angle, this static image looks more like an inflammatory lesion.The third case is a lesion with a neoplastic blockage of the lumen (**Figure 5C**), however, the oozing blood on the surface makes it look more like severe lumen stenosis.During the actual examination of the latter two lesions, doctors can avoid misdiagnosis by changing the angle of fiberoptic bronchoscope and dynamically observing the lesions. **Figure 6** shows 5 cases of benign cases wrongly classified, including 1 case of granulation hyperplasia after tracheotomy, 3 cases of infiltrating lesions after lung infection, and 1 case of old pulmonary tuberculosis with bronchial occlusive changes.Considering that the diagnosis and treatment of patients can only be carried out after fibrobronchoscopy combined with other tumor markers, CT and pathological results, the consequences of missed diagnosis under fibrobronchoscopy are more serious than those of misdiagnosis, which makes the specificity of 0.833 acceptable.

Our study attempted to classify different pathological types of lung cancer, and achieved an overall accuracy of 0.605 in the classification of SCC, AC and SCLC. The fact that there was no difference between the amount of proliferative type and that of infiltrative type in SCLC had a certain impact on the classification results. After screening out SCLC, the classification accuracy of SCC and AC improved, and the accuracy of SCC was still significantly higher than that of AC. The reason lies in lesions of SCC are mainly proliferative, which was the most morphologically different proportion in the study. For this proportion, the feature extraction was more obvious. Additionally, SCC is the main type visible in bronchoscopy. The number of AC in this study was much less than that of SCC, which was not enough for the model to fully learn the subtle features of AC. Another reason is that in order to be as close to the real environment as possible and to ensure the objectivity of our experiment, we tried to include images shooted from different angles around the same lesion, which means images taken far from lesions or in dark light, defocused or blurred ones were also investigated. These images with less satisfying qualities had some impact on the neural network as to learning more subtle surface features of various lung cancer types. Aoki et al. automatically detected erosions and ulcers in wireless capsule endoscopy images based on deep convolutional neural network (29). The lesions were framed by rectangles in the process of image preprocessing, thus it could make feature learning more accurate. Because branch-type bronchial tract has more lumens and more complex structure than digestive tract, the model’s focused area was more likely to be outside the lesion, which could lead to inability to effectively extract subtle details of the lesion for prediction, accounting for errors in classification. In future studies, increasing the number of SCLC and AC to add morphological diversity of lesions, and further screening out images that are not clearly characterized, or applying segmentation algorithms or attention modules to more accurately locate the lesion area are expected to improve accuracy of pathological classification of lung cancer.

Moreover, it took only 30s to diagnose all test set images, and its recognition efficiency was much higher than that of manual work. The final diagnosis performance was as high as that of 2 clinicians with abundant experience in bronchoscopy. The accuracy and sensitivity were significantly higher than 7 other doctors with different experiences. Similarly, Luo et al. developed an AI-assisted diagnostic system for detection of upper gastrointestinal cancer in digestive endoscopy based on multi-center cooperation (30). They compared the results made by the system with those by experts, attendings and interns. It was discovered that the accuracy rate of AI auxiliary diagnosis system in five external test sets was 0.915-0.977, AUC was 0.966-0.990 and the sensitivity was much higher than that of the latter two levels. The data means that if AI-assisted diagnostic model can be successfully applied to the clinic, it can greatly improve efficiency, reduce the workload of endoscopists, assist doctors to improve accuracy and reduce missed diagnosis. It is promising in training primary doctors and being adopted as a remote consultation to aid poor areas where the number of endoscopists is terribly insufficient.

However, there are some limitations in our research. Firstly, the number of benign cases is relatively small. There is a lack of differentiation from benign tumors. The hidden early-stage lung cancer lesions may not be excluded from benign lesions because they are not found by routine examinations. Secondly, the model is developed upon stationary images and cannot guarantee real-time performance required for clinical application. Lastly, all images in this study were retrospectively collected from one bronchoscopy system in a single medical center. The model hasn’t been tested by other hospitals. Therefore, the generalization and stability of the model needs to be further verified.

In the future, we need to obtain larger sample data from more bronchoscopy centers for model training and testing, and we need prospective experiments to test and validate the model more rigorously to improve its stability and applicability. Besides, recording videos during bronchoscopy to train an AI assisted diagnostic model that can be used for real-time judgment during the examination. Finally, since the discovery of early-stage lung cancer can greatly improve survival rate, more bronchoscopy images of early-stage lung cancer should be obtained through multi-center cooperation. We look forward to developing a more excellent AI assisted diagnostic system that can identify early-stage lung cancer.

## 5 Conclusion

In this study, a diagnostic model was constructed for determining benign and malignant lesions in white light bronchoscopy based on deep learning method and classifying pathological types of lung cancer. The performance of this model to distinguish benign and malignant lesions in bronchoscopy is roughly the same as that of experienced clinicians, and the efficiency is much higher than manual work, which verifies the possibility of applying IRT to white light bronchoscopy.

## Data availability statement

The original contributions presented in the study are included in the article/supplementary material. Further inquiries can be directed to the corresponding authors.

## Ethics statement

The study was approved by the Medical Ethics Council for Researchers of the Third Xiangya Hospital affiliated to Central South University. The ethic approved number is FastI 22145. Written informed consent for participation was not required for this study in accordance with national legislation and institutional requirements.

## Author contributions

YD collected the pictures and the patients’ data. YC performed the construction and verification of image recognition model. YD and YC performed the statistical analysis and wrote the manuscript. LX, LW and JZ conceived designed, guided and supervised the study, drafted and revised paper. All authors contributed to the article and approved the submitted version.

## Funding

This study was supported by Fundamental Research Funds for the Central Universities (20720210121), Ministry of Science and Technology of the People’s Republic of China (2021ZD0201900) (2021ZD0201903) and Medical and health Technology Plan Project of Xiamen (3502Z20194021).

## Conflict of interest

The authors declare that the research was conducted in the absence of any commercial or financial relationships that could be construed as a potential conflict of interest.

## Publisher’s note

All claims expressed in this article are solely those of the authors and do not necessarily represent those of their affiliated organizations, or those of the publisher, the editors and the reviewers. Any product that may be evaluated in this article, or claim that may be made by its manufacturer, is not guaranteed or endorsed by the publisher.
